# Risk factors and characteristics influencing humoral response to COVID-19 vaccination in patients after allogeneic stem cell transplantation

**DOI:** 10.3389/fimmu.2023.1174289

**Published:** 2023-05-03

**Authors:** Marie Luise Hütter-Krönke, Adela Neagoie, Igor Wolfgang Blau, Verena Wais, Lam Vuong, Andrea Gantner, Johann Ahn, Olaf Penack, Jacqueline Schnell, Klaus Axel Nogai, Bettina Eberspächer, Maral Saadati, Axel Benner, Lars Bullinger, Hartmut Döhner, Donald Bunjes, Elisa Sala

**Affiliations:** ^1^ Charité – Universitätsmedizin Berlin, Freie Universität Berlin and Humboldt Universität zu Berlin, Department of Hematology, Oncology and Tumorimmunology, Berlin, Germany; ^2^ Department of Internal Medicine III, University Hospital Ulm, Ulm, Germany; ^3^ Department of Microbiology and Hygiene, Labor Berlin- Charité Vivantes GmbH, Berlin, Germany; ^4^ Saadati Solutions, Ladenburg, Germany; ^5^ Division of Biostatistics, Deutsches Krebsforschungszentrum Heidelberg, Heidelberg, Germany; ^6^ German Cancer Consortium (DKTK), Partner Site Berlin, Berlin, Germany

**Keywords:** COVID 19-vaccination, humoral response, allogeneic stem cell transplantation, SARS-CoV-2 antibodies, vaccine, booster, SARS-CoV-2-vaccination

## Abstract

**Introduction:**

Vaccination against severe acute respiratory syndrome coronavirus type 2 (SARS-CoV-2) is approved and recommended for immunocompromised patients such as patients after allogeneic stem cell transplantation (allo-SCT). Since infections represent a relevant cause of transplant related mortality we analyzed the advent of immunization to SARS-CoV-2 vaccination in a bicentric population of allogeneic transplanted patients.

**Methods:**

We retrospectively analyzed data of allo-SCT recipients in two German transplantation centers for safety and serologic response after two and three SARS-CoV-2 vaccinations. Patients received mRNA vaccines or vector-based vaccines. All patients were monitored for antibodies against SARS-CoV2-spike protein (anti-S-IgG) with an IgG ELISA assay or an EIA Assay after two and three doses of vaccination.

**Results:**

A total of 243 allo-SCT patients underwent SARS-CoV-2 vaccination. The median age was 59 years (range 22-81). While 85% of patients received two doses of mRNA vaccines, 10% had vector-based vaccines and 5% received a mixed vaccination. The two vaccine doses were well tolerated with only 3% patients developing a reactivation of graft versus host disease (GvHD). Overall, 72% of patients showed a humoral response after two vaccinations. In the multivariate analysis age at time of allo-SCT (p=0.0065), ongoing immunosuppressive therapy (p= 0.029) and lack of immune reconstitution (CD4-T-cell counts <200/μl, p< 0.001) were associated with no response. Sex, intensity of conditioning and the use of ATG showed no influence on seroconversion. Finally, 44 out of 69 patients that did not respond after the second dose received a booster and 57% (25/44) showed a seroconversion.

**Discussion:**

We showed in our bicentric allo-SCT patient cohort, that a humoral response could be achieve after the regular approved schedule, especially for those patients who underwent immune reconstitution and were free from immunosuppressive drugs. In over 50% of the initial non-responders after 2-dose vaccination, a seroconversion can be achieved by boostering with a third dose.

## Introduction

Severe acute respiratory syndrome coronavirus 2 (SARS-CoV2) is a challenging health issue especially in immunocompromised individuals. The introduction of effective vaccinations, monoclonal antibodies and anti-viral treatment, together with the spread of less hazardous variants has eased the current scenario in many parts of the world (1). However, patients with hematological malignancies, especially after allogeneic stem cell transplantation (allo-SCT), are still at a higher risk of developing severe courses of the disease or of presenting a long-lasting infection, potentially difficult to eradicate (2, 3). These conditions could both directly and indirectly increase the mortality risk associated with SARS-CoV2 infection, especially as compared with the general population. In this context, the promotion of immunization against the virus is of central importance.

Vaccination with newly developed mRNA vaccines (BNT162b2 and mRNA-1273), adenovirus vector-based vaccines (ChAdOx1 and JNJ-78436735) and protein-based vaccines (Nuvaxovid) against SARS-CoV-2 are approved and strongly recommended in hematological patients, also in the post allo-SCT setting. The European Society of Blood and Bone Marrow Transplantation (EBMT) recommends the vaccination for transplanted patients as early as 3 months after allo-SCT in the absence of active acute or chronic graft versus host disease (GvHD) and depending on the actual infectious scenario (4, 5). Full vaccination consists of a 2-dose vaccination of a SARS-CoV2 vaccine at least three weeks apart. The two dose schedule was applied to all patients until September 2021, when the third dose or “booster” vaccination was suggested by the German Vaccination Commission (Ständige Impfkommission, STIKO). A second booster vaccination is already recommended for the immunocompromised or elderly patient group since August 2022 (6, 7). So far, transplanted patients were mostly included in cohorts of hematological malignancies at high-risk for developing no immune response after SARS-CoV-2 vaccination. Furthermore, most of the published cohorts were monocentric and/or with fewer than 100 patients (8–11). The subset of allo-SCT-patients was also often described together with solid organ transplanted patients, even though these patients have other risk factors for not developing seroconversion as compared allo-SCT-patients (12, 13). Reported responses vary between 69-89% after standard immunization in allo-SCT patients after receiving an mRNA vaccine (8–17). A systematic review of the available data regarding the serologic response after two doses of SARS-CoV2 vaccines in the post allo-SCT setting reveals a significantly reduced humoral response rate in this population of immunocompromised patients (18). In addition, data of humoral responses after booster vaccination, especially for initial non-responder allo-SCT patients, are scarce with only small cohorts published (19–23).

Since infections represent a relevant cause of transplant-related mortality and the risk factors impairing the development of a humoral response after allo-SCT are not completely defined, the aim of the present work was to analyze the potential involved predisposing factors for the lack of humoral response after SARS-CoV-2 vaccination in a bicentric population of allogeneic transplanted patients.

## Materials and methods

We performed a bicentric retrospective study evaluating a total of 243 patients undergoing allo-SCT for different hematological diseases and receiving two and three (booster) SARS-CoV-2 vaccinations during the post-transplant follow-up. Patients were transplanted between 1991 and 2021 at the Bone Marrow Transplantation Units of the University Hospitals of Ulm and Berlin (Germany). The study was approved by the local ethic committees on 03/2022. All patients had given written consent before transplant for data collection for future research in accordance with the Declaration of Helsinki. Data cut-off was on 02/2022. We considered as inclusion criteria for the present analysis (1): confirmed diagnosis of malignant and non-malignant hematological disorder requiring transplant; (2) adult age (≥ 18 years); (3) performed allo-SCT from any of the following donors: sibling (SIB), matched unrelated donor (MUD), mismatched unrelated donor (MMUD), haploidentical donor (HAPLO); (4) vaccination against SARS-CoV-2 (at least two doses, third vaccination as booster vaccination was also evaluated). No restriction was applied according to the type of vaccination. All evaluated patients received either mRNA vaccines, like BNT162b2 or mRNA-1273, or the vector-based vaccines ChAdOx1 and JNJ-78436735. We excluded patients with a history of COVID 19 infection or patients presenting uncontrolled acute or chronic GvHD and/or uncontrolled infections from the analysis. All patients were monitored after vaccination at regular time-points for the development of antibodies against SARS-CoV2-spike glycoprotein (anti-S-IgG) with an IgG ELISA assay (Euroimmun) or an EIA Assay (Roche). The first controls were performed at least 14 days after the second vaccination and then again at least 14 days after the booster vaccination. At the time point of data cut off for the present analysis, only a portion of patients received the booster or third vaccination, due to timing issues, but also since the third vaccination was not officially recommended for immunocompromised patients before September 2021 in Germany by the STIKO (6).

### Definitions

We considered specific variables as potential risk-factors implied in determining the development of a proper immunity against SARS-CoV-2 after vaccination: intensity of conditioning regimen, the presence of immunosuppression, the advent of immune reconstitution and the occurrence of GvHD in the previous medical history. An ongoing immunosuppressive treatment was considered as an indirect indicator for controlled GVHD (acute or chronic), especially in case of patients that received the vaccination more than 6 months after allo-SCT. Considering the first variable, conditioning regimen, myeloablative conditioning (MAC) and reduced intensity conditioning (RIC) were defined as previously described (24) Immune reconstitution of T-cells was defined as > 200/µl CD4+ T-cells in peripheral blood at two different time-points measured at least one month apart. The role of B-cells through monitoring of CD19+ cells in blood at regular intervals was also analyzed. We used Immunophenotyping/Fluorescence Activated Cell Sorting (FACS) to determine numbers of B- and T-cells. The analysis was performed on peripheral blood samples. T cells were defined as the CD3+ population, with a further differentiation in CD3+CD4+ and CD3+CD8+, while B cells were defined as the CD19+ population.The presence of immunosuppression was defined as the need for prophylaxis or treatment of GvHD. The GvHD prophylaxis usually consisted of a combination of calcineurin-inhibitors (cyclosporin A or tacrolimus) and mycophenolate mofetil, with the addition at least of corticosteroids in case of GvHD requiring systemic immune suppressive treatment. The presence or the development of GvHD before or after vaccination was analyzed. The staging of acute GvHD (aGvHD) was performed according to the 1994 Consensus Conference on Acute GvHD Grading (25), while the grading of chronic GvHD (cGvHD) was performed according to the NIH Consensus Criteria (26, 27).

### Immunogenity against SARS-CoV-2

We used two different assays in order to measure the humoral response against SARS-CoV2-spike glycoprotein after vaccination in the serum of our patients. The EUROIMMUN test system was based on an ELISA immune assay and delivered semi-quantitative IgG-glycoprotein levels with a cut off for antibody detection above 0,8 U/ml. The sensitivity and specificity were 94,4% and 99,6% respectively (28). Quantitative levels of the IgG S1 glycoprotein were performed by an EIA Roche immune assay with the same cut off of 0,8 U/ml and a sensitivity and specificity of 98,8% and 99,9% respectively (29). In a comparative study both tests showed a similarly high specificity on the same samples without a substantial decrease in diagnostic sensitivity. This analysis offers the basis for the comparability of the results of these two different immune assays (30).

The detection of antibodies above the test-specific level at least 14 days after the second and third vaccination was considered as development of humoral response.

### Statistical analysis

The statistical analysis was performed using the statistical software environment R, version 4.1.3. A descriptive overview for the variables considered in the present study is given by median and range for continuous variables and count and percentage for categorical variables. A multivariable logistic regression model was used to evaluate the effect of explanatory variables on development of a humoral response after vaccination. Odds ratios are reported with 95% confidence intervals. Explanatory factors considered in the multivariable analysis are the following covariates: age at the time of transplant, sex, conditioning regimen, ATG use, T-cell immune reconstitution (CD3+CD4+ cells), B-cell counts (CD19+ cells), ongoing immunosuppressive therapy, and time from transplantation to vaccine. In this last case we evaluated three different time-points for dichotomization in order to identify the more suitable timing for vaccination: 6 months, 12 months and 18 months after allo-SCT.

## Results

### Patient characteristics

We evaluated 243 patients after allo-SCT who underwent SARS-CoV-2 vaccination between 03/2021 and 02/2022. All patients were treated in two stem cell transplantation centers in Germany, Ulm University Hospital (n=146) and Charité Berlin (n=97) and neither had a previously known COVID 19 infection nor an immunization against SARS-CoV2. The median age in our cohort was 59 years (range 22-81), 44% of patients were female. Acute myeloid leukemia was the most common diagnosis leading to allo-SCT accounting for 44% (n=107) of all patients. Myeloproliferative disorders, Myelodysplastic Syndromes, Non-Hodgkin Lymphomas, Acute lymphoblastic Leukemia and others were represented in the cohort in 16%, 15%, 13%, 10% and 2% respectively. The vast majority of patients (95%) had undergone the first allogeneic stem cell transplantation, 4% received the second transplantation and one patient was transplanted three times. The most represented donor type was MUD, accounting for 66% of transplantations, followed by SIB (22%), MMRD (10%) and haploidentical donor (2%). Reduced intensity conditioning was used in 53% of the cases, while 46% were treated with MAC prior to transplant. No patient underwent allo-SCT after a non-myeloablative conditioning. Anti-thymocyte globulin was used in 86% of all transplantations. Immune reconstitution had taken place in 64% of all patients. The median time from transplant to first vaccination was 750 days (range 40-10,566 days) with 27% (n=65) of the patients having been vaccinated within one year after transplantation. Only two patients were vaccinated less than 3 months after allo-SCT, 40 days and 71 days respectively. Both were vaccinated at German vaccination centers in the course of the vaccination campaign for frail people based on their own wish and risk. In these two cases, immune reconstitution was not documented prior to vaccination and no GVHD following SARS-CoV-2-vaccination was observed.

The median Follow up for all patients after the first vaccine was 300 days (range 84-408).

Patients’ characteristics are listed in **Table 1**.

**Table 1:** Patient characteristics (n=243).

Variable	N	%
Sex		
Male	135	56%
Female	108	44%
Age (years)		
Median, range	59	22-81
Diagnosis		
AML	107	44%
MDS	37	15%
ALL	23	10%
MPN	38	16%
NHL	32	13%
other	6	2%
Donor type		
SIB	53	22%
MUD	160	66%
MMUD	24	10%
HAPLO	6	2%
Conditioning Regime		
RIC	129	53%
MAC	114	47%
Vaccination		
BNT162b2	201	83%
mRNA-1273	5	2%
ChAdOx1-S	25	10%
JNJ-78436735	1	0,4%
Mixed Vaccination	11	4,6%
Antibody test (SARS-CoV2-Spike glycoprotein)		
Roche EIA	158	65%
EUROIMMUN ELISA	85	35%
Time from transplant to first vaccination		
median, range (days)	750	40-10.566
Time from second/third vaccination to test		
second vac. (median, range (days))	42	5-159
third vac. (median, range (days))	41	6-151

AML, Acute myeloid leukemia; MDS, Myelodysplastic Syndrome; ALL, Acute lymphatic leukemia; MPN, Myeloproliferative Neoplasia; NHL, Non-Hodgkin Lymphoma; SIB, Sibling Donor; MUD, Matched unrelated Donor; MMUD, Mismatched unrelated Donor; HAPLO, Haploidentical Donor; RIC, Reduced intensity conditioning; MAC, Myeloablative conditioning; EIA, Enzymimmunoassay; ELISA, Enzyme-Linked Immunosorbent Assay; Vac, Vaccination.

### Type of vaccination and time point of vaccination after allo-SCT

Eighty-three % of patients received two doses of BNT162b2, 10% received ChAdOx1-S and 2% were vaccinated with two doses of mRNA-1273 respectively. Only one patient had the single dose of JNJ-78436735 vaccine and finally the rest of the patients (5%) received a mixed vaccination, mostly ChAdOx1-S followed by an mRNA-vaccine. One hundred and nineteen patients (49%) received a third vaccination as booster at the time-point of data cut-off. The third vaccination was performed with BNT162b2 and mRNA-1273 in 88% and 12% of cases respectively. No patient had the adenovirus vector-based vaccination as third vaccination. We evaluated 4 different time points considering the first vaccination after allo-SCT: first vaccination in less than 6 months, which was administered in a minority of patients (n=19, 8%), first vaccination between 6 and 12 months in n=46 cases (19%), first vaccination between 12 and 18 months in n=29 cases (12%) and finally first vaccination at >18 months for 149 patients (61%) respectively.

### Safety profile

Considering the safety profile in our population, we observed a reactivation of chronic GvHD 3 weeks after vaccination in 7 out of 243 patients (3%). Six out of 7 patients developed a reactivation of a previously diagnosed chronic GvHD after the second dose of BNT162b2 vaccination. The other patient showed a reactivation of cGvHD after 2 doses of ChAdOx1-S. In 2 out of 7 cases we had a new onset after an already resolved cGvHD, in most cases (5/7) we observed an aggravation of an ongoing and at the time point of vaccination controlled cGvHD. No patient presented a newly diagnosed form of GvHD, either acute or chronic, that could have been timely associated with the occurrence of SARS-CoV-2 immunization. Only one patient suffered from a perimyocarditis after the second vaccination with BNT162b2, which resolved with a course of anti-inflammatory treatment. The patient did not receive a third vaccination. Other grade 3 or higher toxicities were not observed.

### Serologic response

Overall, 72% of patients showed seroconversion after two vaccinations. In detail, we found a humoral response rate in the cohort from the University Hospital in Ulm of 73% and 70% in the Berlin-Charité cohort. Antibody testing was performed in median 42 days (range 5-159 days) after the second vaccination dose and in median 41 days after the third dose (range 6-151 days). The seroconversion rate increased to 80% in 103 patients that received a third vaccination and were available for antibody testing.

**Figure 1:**
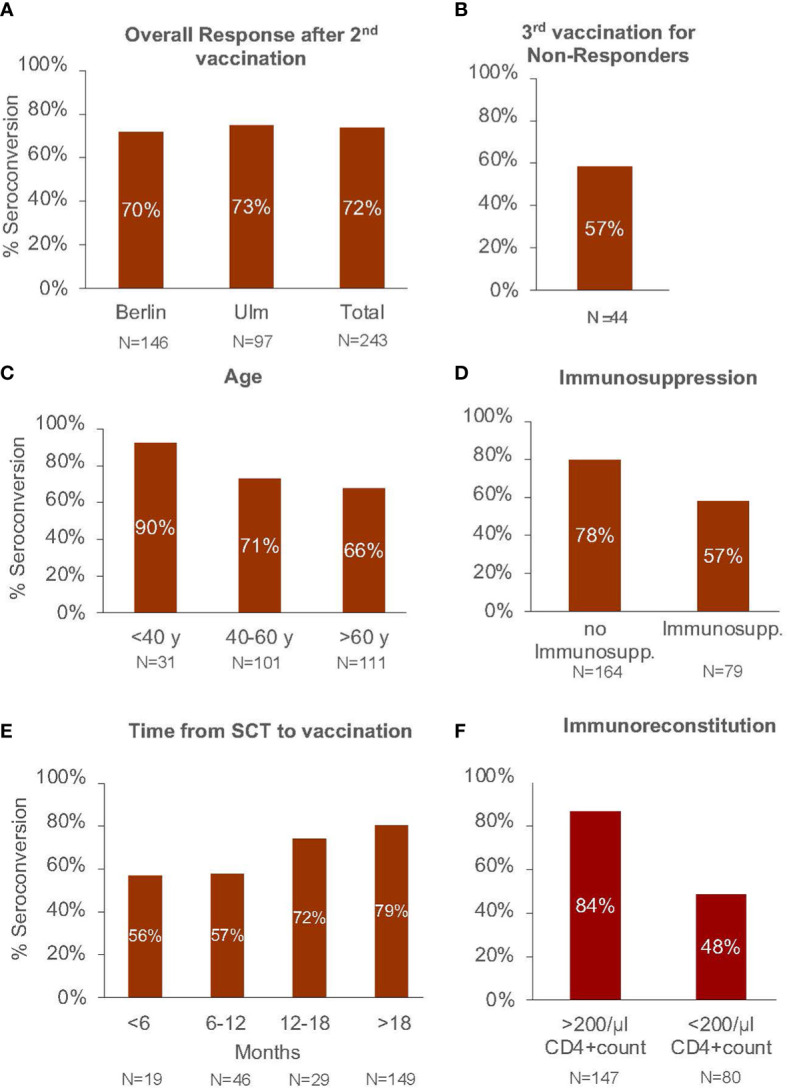
Seroconversation rate in **(A)** the total study cohort after basic vaccination (2 dosages), **(B)** in patients not responding after the second vaccination receiving a third booster vaccination. Seroconversion rate after basic vaccination according to **(C)** patient age, **(D)** ongoing immunosuppressive therapy, **(E)** time interval from allo-SCT to 1. vaccination in months, and **(F)** immunoreconstition after allo-SCT.

In the subgroup of initial non-responders after double vaccination (n=69), 44 patients had a third vaccination, which was not a standard during the data capture time in Germany. Vaccination was performed within a median interval between the second and third dose of 173 days (range, 14-285 days). We found seroconversion in 25 patients of the 44 evaluable patients (57%). A descriptive analysis is shown in **Figure 1**.

### Analysis of risk factors

In the multivariable logistic regression, age at transplantation (odds ratio [OR] = 0.96, p=0.0065) and ongoing immunosuppressive therapy at the time-point of second vaccination (OR = 0.45, p= 0.029) correlated with lower response rates after two vaccine doses. Immune reconstitution was associated with better serologic response (OR 4.28). Sex, intensity of conditioning and the use of ATG in the preparing regimen showed no influence on the development of a serologic response after vaccination.

**Figure 2:**
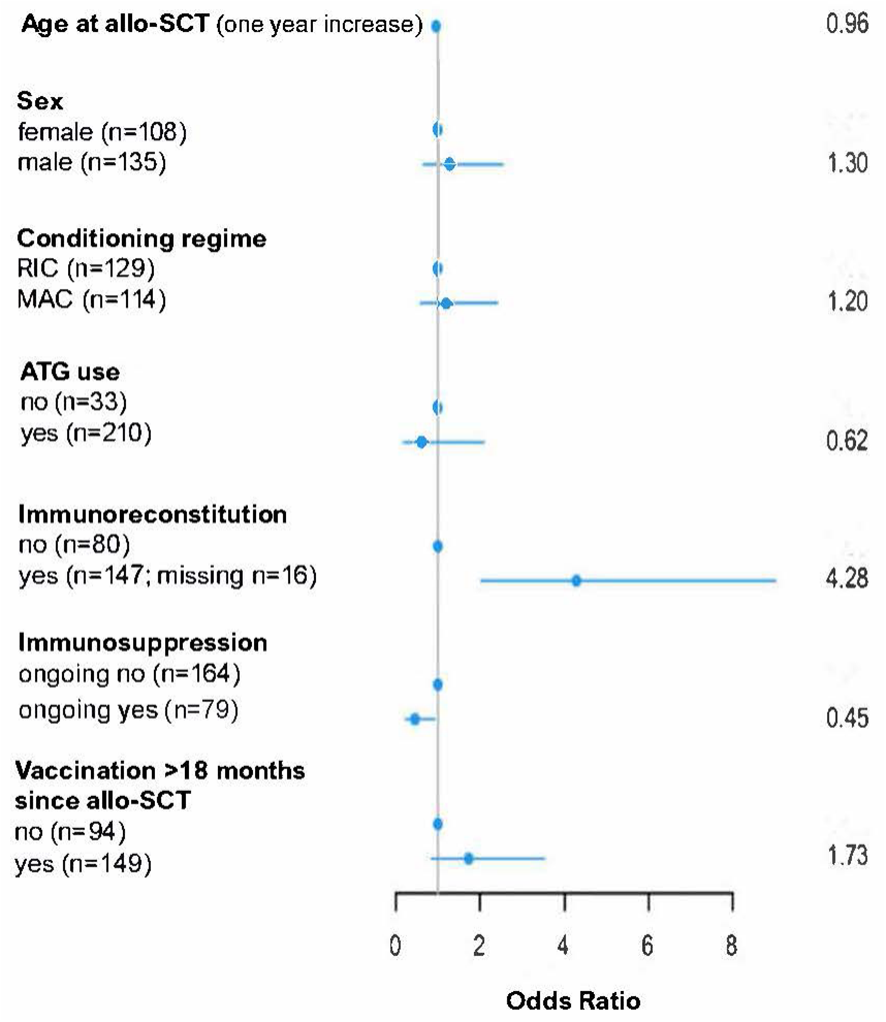
Multivariable logistic regression analysis of variables influencing seroconversation after two SARS-CoV2 vaccinations in allo-SCT patients. allo-SCT, allogeneic stem cell transplantation; ATG, anti-thymocyte globulin; RIC, reduced intensity conditioning; MAC, myeloablative conditioning.

We also analyzed the role of the time interval between allo-SCT and vaccination considering three different time points (+6 months, + 12 months and + 18 months) as potential independent risk factors. No strong correlation was detected between the time after allo-SCT and the probability to get a specific immune response against SARS-CoV-2, even if in the descriptive analysis a trend for a better serologic response rate at late time-points was observed (**Figure 1E**). The results of the multivariable logistic regression analysis are shown in **Figure 2**.

## Discussion

We evaluated a cohort of 243 patients after allo-SCT undergoing SARS-CoV-2 vaccination after transplant from two German transplantation centers. Seroconversion was demonstrated in 72% of patients after two courses of vaccination and increased to almost 80% after the third booster vaccination. These findings are in line with previous studies: humoral response after two vaccinations varied in studies from 69-89% (8–11, 13–15) with the fast majority of humoral response rates between 70-80%. In most studies however, the cohorts were much smaller with fewer than 100 allo-SCT patients. Results on the third vaccination show seroconversion of >=80% (19–23).To our knowledge four comparable published cohorts evaluated a homogeneous data set of cohorts over 100 allo-SCT patients (31, 32). Data from the largest published multicenter study shows the most comparable seroconversion rate of 78% after two vaccinations in 687 transplanted patients from 15 centers in France (31). Pabst et al. showed an almost similar response rate of 81% in their cohort of 167 allo-SCT patients (33). The booster-effect of the third vaccination in our study led to a 56% seroconversion in initial non-responders and is slightly higher than the 41% seroconversion reported in the French study (31). This finding highly supports repetitive booster immunizations in vulnerable patient cohorts. For patients who do not respond to a second booster vaccination a pre-exposition prophylaxis with monoclonal antibodies should be considered (5).

Considering the safety aspects, with a special focus on messenger-RNA vaccines, as adenovirus vector-based vaccines were not largely represented in our population, we observed an advantageous tolerability profile, with only 3% reactivation of cGvHD in our cohort. This is less than the incidence seen in other studies evaluating SARS-CoV2immunization in patients after allo-SCT, even though the median time between allo-SCT and vaccination was longer in the study from Pabst et al, being 3.3 years compared to 2 years in our study (33). Kimura et al. evaluated 95 double vaccinated patients after allo-SCT and found a higher rate of 7,6% GvHD-reactivation, which is well explained by the fact that 54% of the patients were within the first year after allo-SCT (22). In our cohort less than one third of all patients had a time interval between transplantation and vaccination of ≤12 months.

Taking into consideration the humoral response in the post transplantation setting and the potential risk factors accounting for a lack of adequate response, we demonstrated in the multivariate analysis that humoral response to SARS-CoV-2 vaccination was significantly dependent on age at transplantation with worse response rates in the elderly population, the eventually ongoing immunosuppressive therapy and most of all the status of immune reconstitution, especially concerning the T-cell subpopulation of CD3+CD4+ T-cells. We could not find a significant correlation with the development of a humoral immunity after vaccination and the number of CD19+ cells, potentially also due to the only partial completeness of this subset of data in the analyzed cohorts. Other studies found that cellular and humoral immune defects, either because of ongoing immunosuppressive medication or as a consequence of allo-SCT are one of the most significant adverse requisites for not developing humoral response to SARS-CoV-2 vaccination (18).

We also analyzed the role of the time interval between allo-SCT and vaccination, considering different time-points: +6-, + 12- and + 18 months after allo-SCT. In contrast to other studies, we could not find a strong correlation between the time after allo-SCT and the probability of getting a specific humoralresponse against SARS-CoV-2. This potentially suggests that time since transplantation alone might not be an independent factor when adjusted for other important factors, like the number of CD4 T-cells and/or the concomitant administration of immunosuppressive drugs. On the other hand, our cohort probably still does not have a sufficient number of patients to identify a significant difference in serologic response between the time points., Especially when taken in consideration that the majority of patients (61%) in our population was vaccinated after month + 18 post allo-SCT, with the remaining patients heterogeneously distributed in smaller groups in the other time points. To further confirm the reliability of our findings regarding feasibility and humoral response to SARS-CoV-2 vaccination andin respect to potential risk factors, our findings were separately validated in the two different subpopulations of patients transplanted in two different centers in Germany. Limitations of the present analysis are the retrospective design with a heterogeneous population and the absence of a healthy control group. We also used two different tests for determining serologic response and, although the tests are comparable (30), it confers a further element of heterogeneity to our data. Furthermore, prior COVID 19 infection was only determined on a clinical and anamnestic basis.

In summary, the high seroconversion rates and low toxicity observed in our study strongly encourages repetitive vaccinations of allo-SCT patients with highest effects seen after the advent immune reconstitution, in younger patients and patients off immunosuppressive therapy.

## Data availability statement

The raw data supporting the conclusions of this article will be made available by the authors, without undue reservation.

## Ethics statement

The studies involving human participants were reviewed and approved by Ethikkommission der Universität Ulm Helmholtzstraße 20 89081 Ulm, Germany. The patients/participants provided their written informed consent before transplant for data collection for future research in accordance with the Declaration of Helsinki.

## Author contributions

H-KM initiated and designed the study, collected data, wrote the manuscript, and provided patient care. NA, BI, WV, VL, GA, AJ, PO, SJ, and NKA provided patient care. SM and BA performed the statistical analysis, SE designed, and supervised the study, collected data and wrote the manuscript. All authors contributed to the article and approved the submitted version. 

## References

[1] Clinical management of COVID-19: living guideline (2023). Available at: https://www.who.int/publications-detail-redirect/WHO-2019-nCoV-clinical-2023.1 (Accessed April 5, 2023).

[2] Sharma A Bhatt NS St Martin A Abid MB Bloomquist J Chemaly RF. Clinical characteristics and outcomes of COVID-19 in haematopoietic stem-cell transplantation recipients: an observational cohort study. Lancet Haematol (2021) 8:e185–93. doi: 10.1016/s2352-3026(20)30429-4 PMC7816949 33482113

[3] Aydillo T Gonzalez-Reiche AS Aslam S van de Guchte A Khan Z Obla A. Shedding of viable SARS-CoV-2 after immunosuppressive therapy for cancer. N Engl J Med (2020) 383:2586–8. doi: 10.1056/NEJMc2031670 PMC7722690 33259154

[4] Ljungman P Cesaro S Cordonnier C Mikulska M Styczynski J de la Camara R. COVID-19 vaccines (2021). Available at: https://www.ebmt.org/sites/default/files/2021-01/COVID%20vaccines%20version%203.04%20with%20table.pdf.pdf.

[5] Cesaro S Ljungman P Mikulska M Hirsch HH von Lilienfeld-Toal M Cordonnier C. Recommendations for the management of COVID-19 in patients with haematological malignancies or haematopoietic cell transplantation, from the 2021 European conference on infections in leukaemia (ECIL 9). Leukemia (2022) 36:1467–80. doi: 10.1038/s41375-022-01578-1 PMC9053562 35488021

[6] RKI. Epidemiologisches bulletin (2021). Available at: https://www.rki.de/DE/Content/Infekt/EpidBull/Archiv/2021/Ausgaben/39_21.html (Accessed April 5, 2023).

[7] RKI. Epidemiologisches bulletin (2022). Available at: https://www.rki.de/DE/Content/Infekt/EpidBull/Archiv/2022/33/Tabelle.html (Accessed April 5, 2023).

[8] Jiménez M Roldán E Fernández-Naval C Villacampa G Martinez-Gallo M Medina-Gil D. Cellular and humoral immunogenicity of the mRNA-1273 SARS-CoV-2 vaccine in patients with hematologic malignancies. Blood Adv (2022) 6:774–84. doi: 10.1182/bloodadvances.2021006101 PMC8632354 34844263

[9] Greenberger LM Saltzman LA Senefeld JW Johnson PW DeGennaro LJ Nichols GL. Antibody response to SARS-CoV-2 vaccines in patients with hematologic malignancies. Cancer Cell (2021) 39:1031–3. doi: 10.1016/j.ccell.2021.07.012 PMC8295014 34331856

[10] Maneikis K Šablauskas K Ringelevičiūtė U Vaitekėnaitė V Čekauskienė R Kryžauskaitė L. Immunogenicity of the BNT162b2 COVID-19 mRNA vaccine and early clinical outcomes in patients with haematological malignancies in Lithuania: a national prospective cohort study. Lancet Haematol (2021) 8:e583–92. doi: 10.1016/S2352-3026(21)00169-1 PMC8253543 34224668

[11] Dhakal B Abedin S Fenske T Chhabra S Ledeboer N Hari P. Response to SARS-CoV-2 vaccination in patients after hematopoietic cell transplantation and CAR T-cell therapy. Blood (2021) 138:1278–81. doi: 10.1182/blood.2021012769 PMC8332674 34339501

[12] Del Bello A Abravanel F Marion O Couat C Esposito L Lavayssière L. Efficiency of a boost with a third dose of anti-SARS-CoV-2 messenger RNA-based vaccines in solid organ transplant recipients. Am J Transplant Off J Am Soc Transplant Am Soc Transpl Surg (2022) 22:322–3. doi: 10.1111/ajt.16775 PMC8441706 34331842

[13] Li J Ayada I Wang Y den Hoed CM Kamar N Peppelenbosch MP. Factors associated with COVID-19 vaccine response in transplant recipients: a systematic review and meta-analysis. Transplantation (2022) 106:2068–75. doi: 10.1097/TP.0000000000004256 PMC9521391 35761439

[14] Mamez A-C Pradier A Giannotti F Petitpas A Urdiola MF Vu D-L. Antibody responses to SARS-CoV2 vaccination in allogeneic hematopoietic stem cell transplant recipients. Bone Marrow Transplant (2021) 56:3094–6. doi: 10.1038/s41409-021-01466-9 PMC8477622 34584239

[15] Morsink LM van Doesum J Choi G Hazenberg CLE Biswana A Meppelink F. Robust COVID-19 vaccination response after allogeneic stem cell transplantation using post transplantation cyclophosphamide conditioning. Blood Cancer J (2022) 12:6. doi: 10.1038/s41408-021-00605-1 35022420 PMC8754065

[16] Redjoul R Le Bouter A Beckerich F Fourati S Maury S. Antibody response after second BNT162b2 dose in allogeneic HSCT recipients. Lancet Lond Engl (2021) 398:298–9. doi: 10.1016/S0140-6736(21)01594-4 PMC8277189 34270933

[17] Majcherek M Matkowska-Kocjan A Szymczak D Karasek M Szeremet A Kiraga A. Two doses of BNT162b2 mRNA vaccine in patients after hematopoietic stem cell transplantation: humoral response and serological conversion predictors. Cancers (2022) 14:325. doi: 10.3390/cancers14020325 35053487 PMC8773492

[18] Ge C Du K Luo M Shen K Zhou Y Guo K. Serologic response and safety of COVID-19 vaccination in HSCT or CAR T-cell recipients: a systematic review and meta-analysis. Exp Hematol Oncol (2022) 11:46. doi: 10.1186/s40164-022-00299-6 35974381 PMC9380660

[19] Redjoul R Le Bouter A Parinet V Fourati S Maury S. Antibody response after third BNT162b2 dose in recipients of allogeneic HSCT. Lancet Haematol (2021) 8:e681–3. doi: 10.1016/S2352-3026(21)00274-X PMC8415894 34487683

[20] Einarsdottir S Martner A Nicklasson M Wiktorin HG Arabpour M Törnell A. Reduced immunogenicity of a third COVID-19 vaccination among recipients of allogeneic hematopoietic stem cell transplantation. Haematologica (2022) 107:1479–82. doi: 10.3324/haematol.2021.280494 PMC9152965 35236057

[21] Ahmed-Belkacem A Redjoul R Brillet R Ahnou N Leclerc M López-Molina DS. Third early “Booster” dose strategy in France of bnt162b2 SARS-CoV-2 vaccine in allogeneic hematopoietic stem cell transplant recipients enhances neutralizing antibody responses. Viruses (2022) 14:1928. doi: 10.3390/v14091928 36146735 PMC9506309

[22] Kimura M Ferreira VH Kothari S Pasic I Mattsson JI Kulasingam V. Safety and immunogenicity after a three-dose SARS-CoV-2 vaccine schedule in allogeneic stem cell transplant recipients. Transplant Cell Ther (2022) 28:706.e1–706.e10. doi: 10.1016/j.jtct.2022.07.024 PMC9334861 35914727

[23] Watanabe M Yakushijin K Funakoshi Y Ohji G Ichikawa H Sakai H. A third dose COVID-19 vaccination in allogeneic hematopoietic stem cell transplantation patients. Vaccines (2022) 10:1830. doi: 10.3390/vaccines10111830 36366338 PMC9695068

[24] Bacigalupo A Ballen K Rizzo D Giralt S Lazarus H Ho V. Defining the intensity of conditioning regimens: working definitions. Biol Blood Marrow Transplant J Am Soc Blood Marrow Transplant (2009) 15:1628–33. doi: 10.1016/j.bbmt.2009.07.004 PMC2861656 19896087

[25] Przepiorka D Weisdorf D Martin P Klingemann HG Beatty P Hows J. 1994 Consensus conference on acute GVHD grading. Bone Marrow Transplant (1995) 15:825–8. 7581076

[26] Jagasia MH Greinix HT Arora M Williams KM Wolff D Cowen EW. National institutes of health consensus development project on criteria for clinical trials in chronic graft-versus-Host disease: i. the 2014 diagnosis and staging working group report. Biol Blood Marrow Transplant J Am Soc Blood Marrow Transplant (2015) 21:389–401.e1. doi: 10.1016/j.bbmt.2014.12.001 PMC4329079 25529383

[27] Filipovich AH Weisdorf D Pavletic S Socie G Wingard JR Lee SJ. National institutes of health consensus development project on criteria for clinical trials in chronic graft-versus-host disease: i. diagnosis and staging working group report. Biol Blood Marrow Transplant J Am Soc Blood Marrow Transplant (2005) 11:945–56. doi: 10.1016/j.bbmt.2005.09.004 16338616

[28] Beavis KG Matushek SM Abeleda APF Bethel C Hunt C Gillen S. Evaluation of the EUROIMMUN anti-SARS-CoV-2 ELISA assay for detection of IgA and IgG antibodies. J Clin Virol Off Publ Pan Am Soc Clin Virol (2020) 129:104468. doi: 10.1016/j.jcv.2020.104468 PMC7255182 32485620

[29] Elecsys. Anti-SARS-CoV-2 s. diagnostics. Available at: https://diagnostics.roche.com/global/en/products/params/elecsys-anti-sars-cov-2-s.html (Accessed April 6, 2023).

[30] Haselmann V Kittel M Gerhards C Thiaucourt M Eichner R Costina V. Comparison of test performance of commercial anti-SARS-CoV-2 immunoassays in serum and plasma samples. Clin Chim Acta Int J Clin Chem (2020) 510:73–8. doi: 10.1016/j.cca.2020.07.007 PMC7343640 32652161

[31] Maillard A Redjoul R Klemencie M Labussière Wallet H Le Bourgeois A D’Aveni M. Antibody response after 2 and 3 doses of SARS-CoV-2 mRNA vaccine in allogeneic hematopoietic cell transplant recipients. Blood (2022) 139:134–7. doi: 10.1182/blood.2021014232 PMC8616709 34818411

[32] Piñana JL López-Corral L Martino R Montoro J Vazquez L Pérez A. SARS-CoV-2-reactive antibody detection after SARS-CoV-2 vaccination in hematopoietic stem cell transplant recipients: prospective survey from the Spanish hematopoietic stem cell transplantation and cell therapy group. Am J Hematol (2022) 97:30–42. doi: 10.1002/ajh.26385 34695229 PMC8646900

[33] Pabst C Benning L Liebers N Janssen M Caille L Speer C. Humoral responses and chronic GVHD exacerbation after COVID-19 vaccination post allogeneic stem cell transplantation. Vaccines (2022) 10:330. doi: 10.3390/vaccines10020330 35214787 PMC8876761

